# Efficacy of a Dual‐Action SPF 50+/PA++++ Sunscreen: A Multi‐Targeted Approach to Photoprotection and Skin Barrier Recovery

**DOI:** 10.1111/jocd.71253

**Published:** 2026-10-22

**Authors:** Jiamin Gu, Yue Ying Fu, Vanessa Koh, Hiroki Fujita, Jessica Wang, Sheila Ching Chua, Yuan Yuan Duan, Daiki Arai, Junchao Zhang, Mao Kasahara, Wei Lai

**Affiliations:** ^1^ Kenvue (Shanghai) Healthcare Co. Ltd Shanghai China; ^2^ Kenvue Singapore Pte. Ltd. Singapore; ^3^ JNTL Consumer Health K.K. Tokyo Japan; ^4^ Department of Dermatology The Third Affiliated Hospital of Sun Yat‐sen University Guangzhou China

**Keywords:** antioxidant, barrier recovery, photoaging, sunscreen, ultraviolet radiation

## Abstract

**Background:**

As sunscreens do not completely block ultraviolet radiation, chronic UVR exposure causes cumulative photodamage, increasing premature photoaging, and skin cancer risk. Hence, an optimal sunscreen should incorporate dual benefits, going beyond photoprotection while actively supporting skin barrier recovery post sun‐exposure.

**Aim:**

To evaluate the broad‐spectrum photoprotective and barrier repair efficacy of a dual‐action sunscreen with Smart Layers Technology. Primary outcomes included photoprotection and barrier repair; secondary outcomes included pigmentation and skin tone improvement.

**Methods:**

Photoprotection was evaluated in vivo, single center, evaluator blinded, self‐controlled study. Antioxidant activity was examined in an ex vivo human skin model under UVR‐induced oxidative stress. Barrier repair was studied in a randomized controlled trial and in vitro UVR‐induced epidermal barrier model, measuring filaggrin, loricrin, and claudin‐1. Pigmentation and skin tone were assessed in vivo UVR‐induced pigmentation model.

**Results:**

The dual‐action sunscreen demonstrated high photoprotective efficacy, with a mean SPF of 56.2, UVA protection factor of 18.9, and 63.9% water resistance. Reactive oxygen species were reduced by 57.74%, demonstrating antioxidant effects. Post UVR‐irritated skin showed significant improvement in skin hydration and accelerated normalization of transepidermal water loss (both *p* < 0.001). Expression of key barrier proteins, filaggrin, loricrin, and claudin‐1, increased by 273.68%, 139.39%, and 167.57% respectively versus negative control. Skin brightness improved significantly (L*value; *p* < 0.001) with a significant reduction in pigmentation (a*value; *p* < 0.01) versus negative control. Skin melanin index and individual typology angle improved significantly (*p* < 0.05) compared to negative control.

**Conclusion:**

The dual‐action sunscreen addresses photodamage prevention and barrier recovery through broad‐spectrum photoprotection and UVR‐exposure skin barrier repair.

## 1 Introduction

Regular sunscreen use is essential in mitigating both acute and chronic skin damage caused by ultraviolet (UV) exposure, thereby reducing the risk of photoaging and skin cancers [1]. The World Health Organization recommends comprehensive photoprotection, including limiting midday sun exposure, wearing protective clothing, and applying broad‐spectrum sunscreen (SPF 30 or higher) to exposed skin, with reapplication every 2 h during activities involving sweating or swimming [2]. Sunscreen use is no longer limited to planned sun exposure. Dermatologists increasingly advocate for proactive, year‐round sunscreen use, as UV radiation is present even on cloudy days [3]. Modern sunscreens not only prevent UV‐induced sunburn but also protect against photoaging, dyspigmentation, DNA damage, and photo‐carcinogenesis [4].

UV exposure to the skin is known to cause inflammation, oxidative stress, and DNA damage [5]. Evidence also suggests that photodamage begins to accumulate at an early age, with some indicating that 50% of lifetime UV exposure occurs by the age of 20 [6]. For example, among the Japanese population, the first signs of photoaging are usually solar lentigines; these could be observed among individuals age 20, with fine wrinkles and benign skin tumors appearing after age 30 and 35, respectively [7]. A recent study suggests that repeated UV exposure can alter skin homeostasis without causing visible erythema, and that the underlying molecular alterations may not be fully preventable [8]. Bienkowska et al. demonstrated that though SPF 30 sunscreen mitigates UV‐induced epigenetic changes, minor molecular perturbations in cellular repair persist. These alterations increase the risk of cumulative photodamage driving structural and immunological changes that impair skin barrier function [8]. Therefore, it is crucial that an optimal sunscreen should not only provide immediate photoprotection but also repair existing cumulative photodamage and improve overall skin health.

Additionally, phototypes and ethnicity are important determinants of cutaneous response to UV exposure; hence, personalized photoprotection is important as well [9, 10, 11]. Asian skin exhibits unique structural and functional characteristics such as relatively thinner epidermis and a denser dermis. The thinner stratum corneum and weaker barrier allow greater UV penetration and faster onset of irritation, resulting in higher UV sensitivity [12]. Asian skin is also particularly prone to pigmentary disorders, with a reported melasma prevalence of 30%–50% [12, 13, 14, 15]. Despite a lower incidence of skin cancer in Asians, the analysis of melanoma cases from the Surveillance, Epidemiology, and End Results (SEER) database from 1975 to 2020 indicates that melanoma rates in Asian populations in the United States are rising, with an estimated incidence of 1.3 per 100 000 persons. Furthermore, Asian melanoma patients experience a 27% higher mortality rate than Caucasians, mainly due to limited awareness and late‐stage presentation [13, 14, 15, 16]. Inadequate photoprotective behaviors and misconceptions about sunscreen application further exacerbate this risk [17]. Despite these issues, studies on photoprotection in Asian skin are limited [13, 18].

As photodamage starts accumulating from the early twenties, repeated UV exposure over time progressively compromises the structural and functional integrity of the skin barrier. However, most traditional sunscreens typically prioritize prevention of UV penetration and erythema and often overlook the crucial need for skin barrier repair and restoration. To address these gaps in photoprotection, the present study aimed to evaluate the efficacy of a dual‐action sunscreen specifically among Asian populations. The dual‐action sunscreen incorporates Smart Layers Technology designed to deliver both photoprotection and post‐exposure barrier repair. Five complementary studies were conducted to assess the primary outcomes of broad‐spectrum photoprotection and post sun exposure skin barrier repair. Secondary outcomes included evaluation of additional benefits such as pigmentation and skin tone improvement. This approach responds to the unique ethnic and phototype needs of Asian skin prone to hyperpigmentation and offers a comprehensive strategy to improve photoprotection, barrier recovery, and overall skin health.

## 2 Methods

### 2.1 Smart Layers Technology

The Smart Layers Technology incorporated in the dual‐action sunscreen combines photostable, broad‐spectrum UV protection with potent skincare actives to protect, hydrate, and repair the skin. The Smart Layers Technology does not refer to any specific ingredients; rather, it is the formulation technology that achieves UV protection and dermatological benefits through the different layers. The outer layer focuses on stable, broad‐spectrum photoprotection using a blend of chemical and physical UV filters to protect against UVA and UVB (labeled as SPF 50+). The inner layer delivers targeted skincare benefits with active ingredients: phenylethyl resorcinol, sclareolide, Panthenol (vitamin B5), mini hyaluronic acid (HA), and vitamin C derivatives.

### 2.2 Study Design and Outcome Framework

The efficacy of the dual‐action sunscreen was evaluated across five complementary in vivo, ex vivo, and in vitro studies assessing UVR‐damage prevention and post‐exposure skin recovery (Table 1 and S1–S5). The studies were specifically conducted in participants with existing photodamage and designed in UVR‐damaged skin models to establish that significant photodamage had already accumulated prior to the application of dual‐action sunscreen. The indicators of photodamage and skin barrier function were measured at baseline and after sunscreen application to substantiate the immediate photoprotective and long‐term barrier restorative efficacy of the dual‐action sunscreen.

**TABLE 1:** Overview of in vivo, ex vivo, and in vitro studies to evaluate the primary outcomes of photoprotection and skin barrier repair, and the secondary outcomes of pigmentation and skin tone improvement for the dual‐action sunscreen.

Study number	Model	Study design/assay	UV exposure	Comparator	Endpoint	Outcome measure
Primary outcomes						
Study 1	In vivo	Single‐center, evaluator‐blind, self‐controlled clinical study	UVA + UVB	Unprotected skin	Photoprotection	Photoprotective efficacy following UV exposure (SPF, UVAPF, water resistance)
Study 2	Ex vivo	Human skin model under UVR‐induced oxidative stress	UVA + UVB	Blank, negative, positive controls	Photoprotection (antioxidant activity)	Reduction in UVR‐induced oxidative stress (ROS inhibition)
Study 3	In vivo	Randomized controlled clinical trial (*n* = 35)	UV‐induced irritation	Blank/untreated skin sites	Skin barrier repair	Post‐UV skin barrier recovery (skin hydration, TEWL, erythema)
Study 4	In vitro	UVR‐induced human epidermal barrier‐impact model	UVB	Negative and positive controls	Skin barrier repair	Filaggrin, loricrin, and claudin‐1 expression
Secondary outcomes						
Study 5	In vivo	UVR‐induced pigmentation model	Repeated UV	Negative control	Pigmentation improvement	Pigmentation (melanin index) and skin tone improvement (L*, a*, ITA°)

Primary outcomes included photoprotection and skin barrier repair. Photoprotection and antioxidant efficacy were evaluated in Study 1: an in vivo, single‐center, evaluator‐blind, self‐controlled clinical trial, and Study 2: an ex vivo human skin model assessing UVR‐induced oxidative stress. Skin barrier repair efficacy was evaluated in Study 3: a randomized clinical trial (*n* = 35) assessing skin hydration, transepidermal water loss, and clinical tolerance following controlled UV‐induced irritation; and Study 4: an in vitro UVR‐induced human epidermal barrier impact model, quantifying expression of filaggrin (FLG), loricrin (LOR), and claudin‐1 (CLDN1). The secondary outcome, pigmentation and skin tone improvement, was evaluated in Study 5 using an in vivo UVR‐induced pigmentation model.

### 2.3 Photoprotection: Study 1—In Vivo Photoprotection Assessment

The Sun Protection Factor (SPF), Ultraviolet A Protection Factor (UVAPF) and water resistance of the dual‐action sunscreen were evaluated via an in vivo single center, evaluator‐blind, self‐controlled protocol in accordance with International Organization for Standardization (ISO) methods [19]. In accordance with ISO 24444:2019, ISO 18861:2020 and ISO 16217:2020, and ISO 24442:2022 methodologies, untreated skin sites on each participant served as the negative control. Positive control was not used as aligned with ISO protocol. Instead, a reference sunscreen specified in the relevant ISO standard was included to verify the validity and reproducibility of the test system. Informed consent was obtained from all participants, and the study was approved by the Guangdong Daily Chemical Ethics Committee.

Ten healthy adults (18–60 years; ITA° ≥ 28.0) were enrolled for SPF and water resistance assessment; subjects with ITA° values between 28 and 43 were included for UVA protection evaluation. The sample size of 10 subjects was selected in accordance with the requirements of ISO 24444 and ISO 24442, which specify a minimum of 10 valid evaluable subjects for SPF and UVA‐PF determination. The intrascapular back was exposed to UVA + UVB using a solar simulator (300‐watt xenon arc; 290–400 nm). Minimal erythemal dose (MED) was recorded 20 ± 4 h post‐irradiation. SPF was calculated as the ratio of MED on sunscreen‐protected to unprotected skin. The mean SPF values were expressed as the arithmetic mean of valid measurements truncated to one decimal place. The SPF met ISO statistical validity criteria when the 95% confidence interval (CI) fell within ± 17% of the mean, calculated as follows:

95%CI[SPF]=SPF±0.17×SPF

Water resistance was assessed after two 20‐min immersion cycles. Water‐resistant SPF (SPFWR) was calculated from post‐immersion MED ratios. Percentage of water resistance was determined by comparing post‐ and pre‐immersion SPF values, expressed as a mean with unilateral 90% CI, calculated per formula below:^1^

$$90\%CI\;\left[\text{mean}\%\mathrm{WR}\right]=\text{mean}\%WR_{i}-d$$

of which d is calculated as follows:

$$d=t_{u}\times s/\sqrt{\;n}$$

UVA protection was assessed by measuring the minimal persistent pigment darkening dose (MPPDD) 2–24 h after irradiation. Individual UVAPF values were calculated as the ratio of protected to unprotected MPPDD, with mean UVAPF values truncated to one decimal place. UVAPF measurements met statistical validity criteria when the 95% CI fell within ± 17% of the mean, calculated as follows:

95%CI[UVAPF]=UVAPF±0.17×UVAPF

### 2.4 Photoprotection: Study 2—Ex Vivo Antioxidant Evaluation

An ex vivo human skin model was used to evaluate the antioxidant effect of the dual‐action sunscreen by measuring changes in reactive oxygen species (ROS) levels after UV exposure. Skin explants were transferred into 6‐well culture plates and maintained in 3.7 mL of culture medium per well at 37°C with 5% CO_2_, with daily medium changes.

After 2 days of model cultivation, the explants were divided into four groups: Blank control (BC), negative control (NC), positive control (PC), and sample with test product (test). Only NC, PC, and test groups received irradiation [UVA (30 J/cm^2^) and UVB (50 mJ/cm^2^)]. PC and test groups received the first administration at 9 am. PC received culture medium containing Vitamin C 100 μg/mL and Vitamin E 7 μg/mL. The PC (Vitamin C + Vitamin E) was used as baseline reference to validate the operationality of the antioxidant assay, rather than as a direct vehicle‐matched comparator. The UV‐stimulated tissue without any treatment in the NC group represented baseline oxidative damage. The assay was designed for quantitative evaluation of relative ROS reduction under identical UV‐induced oxidative stress conditions. The test group received 2 mg/cm^2^ of dual‐action sunscreen (test product), followed by irradiation, 4‐h incubation, and reapplication. At 5 PM, all skin explants samples were wiped off and cultured, and the steps were repeated for 4 days. Immunofluorescence was assessed using a DCFH‐DA (2′,7′‐dichlorodihydrofluorescein diacetate) probe to detect ROS, and fluorescence intensity was visualized under a microscope.

ROS inhibition rate (%) was calculated as per the equation:

$$\text{Inhibition rate}\;\left(\%\right)=\frac{\text{Negative control group}-\text{Test group}}{\text{Negative control group}}\times 100\%$$

Data were analyzed in GraphPad Prism (Mean ± SD) using two‐tailed *t*‐tests (*p* < 0.05 significant).

### 2.5 Barrier Repair: Study 3—Randomized Clinical Trial

A single‐center, evaluator‐blinded, randomized clinical trial (RCT) evaluated the barrier repair efficacy of the dual‐action sunscreen after controlled UV irritation. The study protocol and the informed consent were approved by the Ethics Committee of Guangdong Daily Chemical Industry. Thirty‐eight subjects aged 26–60 years old were enrolled, of which 35 completed the study. The sample size was determined based on the intra‐individual paired design recommendations from the Safety and Technical Standards for Cosmetics (2022), and findings from a preliminary pilot study. The paired design minimizes inter‐subject variability and enhances statistical sensitivity for detecting treatment‐related differences in skin hydration, transepidermal water loss (TEWL), and erythema. Testing sites in each participant were randomized either to the test group (with application of dual‐action sunscreen) or control group (vehicle only) with evaluators blinded to treatment allocation. UV energy was applied on four testing sites on the skin at the back area to induce controlled irritation. The dual‐action sunscreen was applied 2 h post UV irritation followed by once daily application for 14 days. Instrumental analysis including assessment of skin hydration (Corneometer), transepidermal water loss (TEWL) (Tewameter), and erythema analysis (Mexameter); and tolerance evaluation by clinical dermatological assessment were conducted on Day 0 (D0 pre‐UV), D0T2h (2 h post‐UV), Day 1, Day 2, Day 7, and Day 14.

Skin hydration, TEWL, and erythema index were expressed as mean ± standard deviation and analyzed for both intra‐group and inter‐group differences across timepoints. Intra‐group comparisons evaluated changes from baseline (D0) and from 2‐h post‐UV exposure (D0T2h) using paired statistical tests. The inter‐group comparisons assessed differences in change scores between the dual‐action sunscreen and blank control sites at each follow‐up visit (D1, D2, D7, and D14). Statistical analyses were selected according to the type and distribution of the study variables. Instrument‐based measurements, including skin hydration, TEWL, and erythema index, were analyzed as continuous variables. Paired‐samples *t*‐tests were used for normally distributed data, whereas Wilcoxon signed‐rank tests were applied when normality assumptions were not met. Dermatological clinical tolerance assessments (e.g., edema, comedone, papules, and scaling) were analyzed using non‐parametric methods. The significance thresholds were marked at *p* < 0.05, *p* < 0.01, and *p* < 0.001.

### 2.6 Barrier Repair: Study 4—Quantifying Key Structural Proteins in UVB‐Damaged Epidermis Model

The skin barrier repair efficacy of the dual‐action sunscreen was tested on a UVB‐damaged EpiKutis 3D epidermal model, quantifying changes in FLG, LOR, and CLDN1 levels. The tissues were assigned to four groups: blank control (BC), negative control (NC), positive control (PC), and sample with test product (test). WY14643 (pirinixic acid), a peroxisome proliferator activated receptor alpha (PPARα) agonist, served as the positive control and reference standard for barrier repair [20]. All groups except BC received UVB irradiation at 600 mJ/cm^2^. After irradiation, the PC was treated with culture medium containing 50 μM concentration of pirinixic acid. The test sample was treated with 2 mg/cm^2^ of the dual‐action sunscreen (test product), incubated for 8 h, cleaned and incubated for a further 16 h.

To prepare the epidermal model tissues for immunofluorescence imaging, reconstructed human epidermal skin model (EpiKutis) tissues were affixed with 4% paraformaldehyde, followed by paraffin‐embedding and tissue sectioning onto microscope slides. Slides were deparaffinized through a series of fresh xylene and ethanol washes. The paraffin sections were then soaked in sodium citrate antigen retrieval solution for antigen retrieval, followed by three washes in phosphate‐buffered saline (PBS) for 5 min each. The tissue sections were then stained with the secondary antibodies for 60 min at 37°C to prevent non‐specific binding before overnight incubation with primary antibodies at 4°C. Slides were stained with mouse monoclonal antibodies against human filaggrin (Abcam) at a 1:200 dilution; rabbit polyclonal antibodies against human claudin‐1 (Proteintech) at a 1:200 dilution; and rabbit monoclonal antibodies against human loricrin (Abcam) at a 1:500 dilution.

Following the second antibody incubation, slides were washed with PBS, and Hoechst solution was added to each slide and incubated at room temperature for 5 min for nuclear staining. Intensity of immunostaining was estimated with the use of a fluorescence microscope (Olympus B × 43) within 24 h.

Post‐treatment tissues were processed for immunofluorescence analysis of FLG, LOR, and CLDN1 and measured with integrated optical density (IOD). Fluorescence was measured using antibody‐based immunofluorescence, and fluorescence intensity was visualized under a fluorescence microscope.

The improvement rate of key structural proteins was calculated as:

$$\text{Improvement rate}\;\left(\%\right)=\frac{\text{Test group}-\text{Negative control group}}{\text{Negative control group}}\times 100\%$$

Statistical analysis was performed using GraphPad Prism, with results expressed as mean ± SD. Comparisons between groups were conducted using two‐tailed *t*‐tests to determine statistical significance, with *p* < 0.05 considered significantly different.

### 2.7 Pigmentation and Skin Tone Improvement: Study 5—In Vivo UV‐Induced Skin Darkening Model

Pigmentation and skin tone improvement of the dual‐action sunscreen were assessed in an ultraviolet‐induced human skin pigmentation model including 30 adults with baseline Individual Typology Angle (ITA°) values at test sites ranging from 32.59° to 40.96°. Non–sunexposed thigh areas (≥ 0.5 cm^2^) were irradiated using a light‐simulating instrument once daily for four consecutive days, followed by a four‐day pigmentation development period without treatment. On Day 5 postirradiation, sites with uniform pigmentation were randomized to either negative control (NC) or dual‐action sunscreen (denoted as test). Skin color was assessed at baseline and after 1, 2, 3, and 4 weeks using visual evaluation and instrumental measurements, including individual typography angle (ITA°), melanin index (MI), lightness (L*), and redness (a*).

## 3 Results

### 3.1 Primary Outcomes: Studies 1,2, 3 and 4

#### 3.1.1 Photoprotection: Study 1—Dual‐Action Sunscreen Demonstrates High SPF and Broad Spectrum Photoprotection

The dual‐action sunscreen (test group) demonstrated high UVA + UVB protection with a mean SPF of 56.2 (95% CI: 53.1–59.3), fulfilling the ISO ± 17% statistical validity criteria. The observed water‐resistance retention (63.9%, 90% CI: 60.8%) exceeded the ISO acceptance criterion of 50% required for water‐resistant labelling. These findings indicate that substantial SPF protection was maintained after the standardized water immersion challenge and support the practical effectiveness of the sunscreen under conditions of water exposure. The dual‐action sunscreen yielded a mean UVAPF of 18.9 (95% CI: 18.0–19.8) (Table 2).

**TABLE 2:** In vivo assessment of sun protection factor (SPF), UVA protection factor, and water resistance of dual‐action sunscreen formulation.

SPF/UVAPF/WR	Average	SD
SPF	56.2 (95% CI 53.1–59.3)	4.3
UVA protection factor	18.9 (95% CI 18.0–19.8)	1.3
Percentage of water resistance (40 min immersion) (% WR)	63.9% (90% CI 60.8%)	7.1

*Note:* In vivo SPF (*n* = 10), UVAPF (*n* = 10), and percentage water resistance (*n* = 10) data. Data are presented as mean (95% confidence interval [CI]) and standard deviation (SD), with percentage water resistance data presented as mean unilateral 90% CI. All parameters were evaluated according to ISO 24444:2019 and ISO 24442:2022 following standardized immersion procedures.

Abbreviations: CI, confidence interval; SD, standard deviation; SPF, sun protection factor; UVAPF, ultraviolet A protection factor.

#### 3.1.2 Photoprotection: Study 2—Dual‐Action Sunscreen Demonstrates Antioxidant Effect

UV irradiation increased ROS levels in the negative control compared with the blank control (IOD 5.04 vs. 1.00). Treatment with dual‐action sunscreen (test group) resulted in a significant reduction in ROS relative to the negative control (IOD: 2.13), corresponding to 57.74% inhibition (*p* < 0.01 vs. negative control). The positive control, which served as a benchmark for ROS inhibition, resulted in highest ROS inhibition (77.38%) (Table 3). Representative fluorescence images are shown in Figure 1.

**TABLE 3:** Quantification of reactive oxygen species levels by integrated optical density in an ex vivo human skin model following UV exposure.

Group	Relative IOD	Standard deviation (SD)	Inhibition vs. NC
Blank control (BC)	1.00	0.019	—
Normal control (NC)	5.04	0.24	—
Positive control (PC)	1.14	0.10**	77.38%
Test group (with dual‐action sunscreen)	2.13	0.22**	57.74%

*Note:* Reactive oxygen species (ROS) data are presented as mean (SD). ROS levels were quantified following UVA and UVB irradiation. Inhibition rate (%) was calculated relative to the negative control (NC).

Abbreviations: BC, blank control; NC, negative control; PC, positive control.

** *p* < 0.01 vs. NC.

**FIGURE 1:**
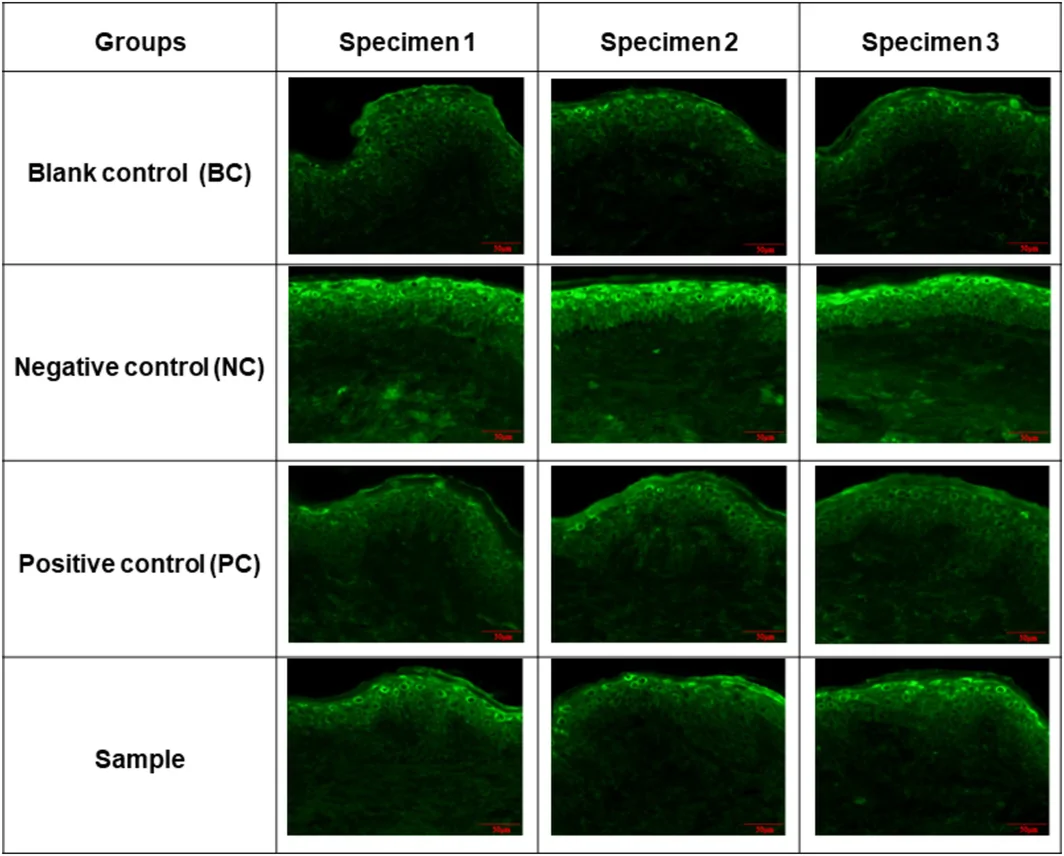
Fluorescence imaging of ROS levels in ex vivo human skin following UV irradiation. ROS generation was visualized via green fluorescence. A reduction in fluorescence intensity indicates lower ROS intensity.

#### 3.1.3 Barrier Repair Study 3—Dual‐Action Sunscreen Demonstrates Barrier Recovery With Improved Skin Hydration and Reduced Water Loss and Erythema

Skin hydration decreased immediately following UV exposure in both groups. In the test group (with dual‐action sunscreen), hydration declined relative to baseline (D0) at, D1, and D2, with a significant recovery and increase above baseline at D7 and D14 (*p* < 0.01) (Figure 2). When compared with D0T2h, hydration values were significantly higher at D7 and D14 (*p* < 0.01) (Figure 3). D0 values were determined to establish baseline hydration prior to UV exposure, while D0T2h values were used to measure UV‐induced skin injury and evaluate immediate photoprotective and hydrating efficacy of the dual‐action sunscreen. In contrast, the blank control group, showed decreased hydration at all post‐UV timepoints through Day 14, with no recovery to baseline levels (Figures 2 and 3). Inter‐group comparisons demonstrated significantly greater improvement in hydration in the test group compared with the blank control at D2, D7, and D14, when compared to both baseline and D0T2h (*p* < 0.001) (Table 4).

**FIGURE 2:**
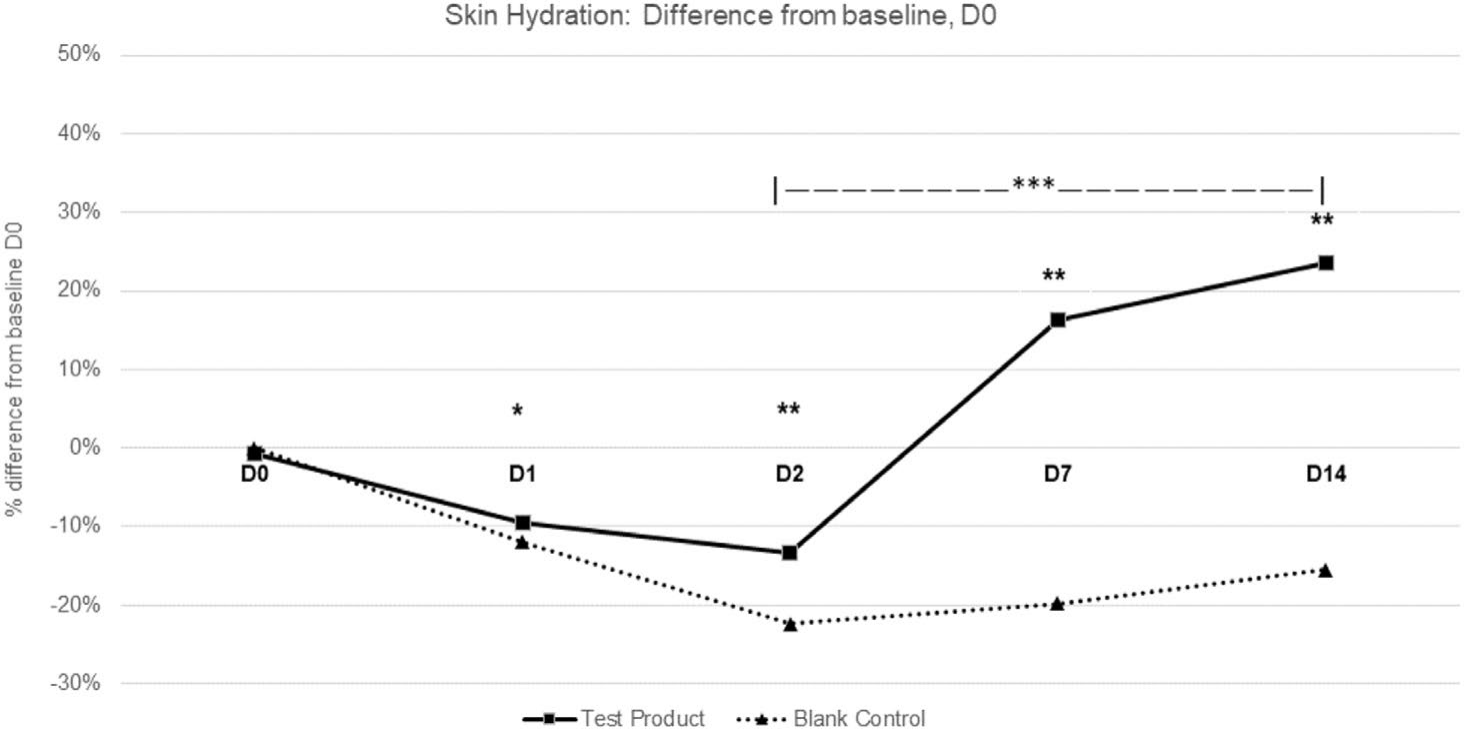
Intra‐group and inter‐group comparison of skin hydration changes from baseline (D0) over a 14‐day period in a randomized clinical study (*n* = 35). Percentage difference in skin hydration relative to baseline (D0) over 14‐days was measured using a Corneometer. Data represent the mean percentage change from baseline (D0) for test product and blank control. *p < 0.05, **p < 0.01 vs. baseline (D0) indicate statistically significant differences in intra‐group comparisons; ***p < 0.001 vs. blank control indicates statistically significant differences between test product and blank control across the treatment period (D2–D14) in inter‐group comparisons.

**FIGURE 3:**
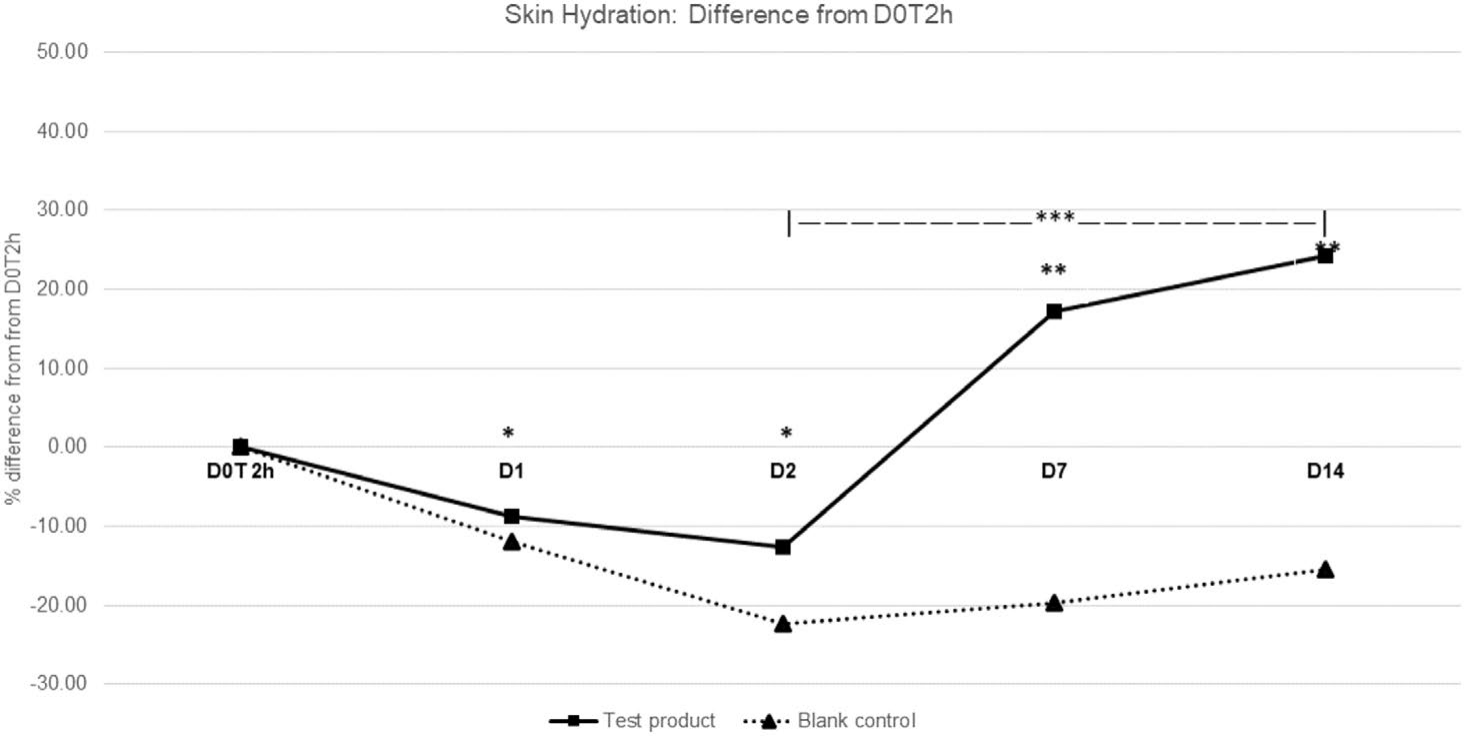
Intra‐group and inter‐group comparison of skin hydration changes from 2‐h post‐UV exposure (D0T2h) over a 14‐day period in a randomized clinical study (*n* = 35). Percentage difference in skin hydration relative to 2‐h post‐UV exposure (D0T2h) over 14 days was measured using a Corneometer. Data represent the mean percentage change in skin hydration from 2‐h post‐UV exposure (D0T2h) for test product and blank control. *p < 0.05, **p < 0.01 vs. baseline (D0) indicate statistically significant differences in intra‐group comparisons; ***p < 0.001 vs. blank control indicates statistically significant differences between test product and blank control across the treatment period (D2–D14) in inter‐group comparisons.

**TABLE 4:** Inter‐group comparison of skin hydration changes from baseline (D0) over a 14‐day period in a randomized clinical study (*n* = 35).

Skin hydration	D0T2h	D1	D2	D7	D14
*p*‐value (vs. D0) test product vs. blank control	0.720	0.340	< 0.001***	< 0.001***	< 0.001***
*p*‐value (vs. D0T2h) test product vs. blank control	N/A	0.160	< 0.001***	< 0.001***	< 0.001***

*Note:* Skin hydration was assessed using a Corneometer. Values shown are *p*‐values comparing the test product with the blank control at each timepoint.

Abbreviations: D0, baseline (pre‐UV exposure); D0T2h, 2 h post‐UV exposure; D1, Day 1; D14, Day 14; D2, Day 2; D7, Day 7.

*** *p* < 0.001 indicates statistically significant inter‐group differences.

TEWL increased after UV exposure in both groups. Compared to D0, TEWL in the test group increased at D0T2h, D1, followed by a significant reduction at D7 and D14 (*p* < 0.01) (Figure 4). Similar trends were observed in comparison with D0T2h (*p* < 0.01) (Figure 5). In contrast, the blank control group showed elevated TEWL across all timepoints, with a slower decline than the test group. Inter‐group comparison showed significantly greater TEWL reduction in the test group versus the blank control at D2, D7, and D14, both relative to baseline and D0T2h (*p* < 0.001) (Figures 4 and 5) (Table 5).

**FIGURE 4:**
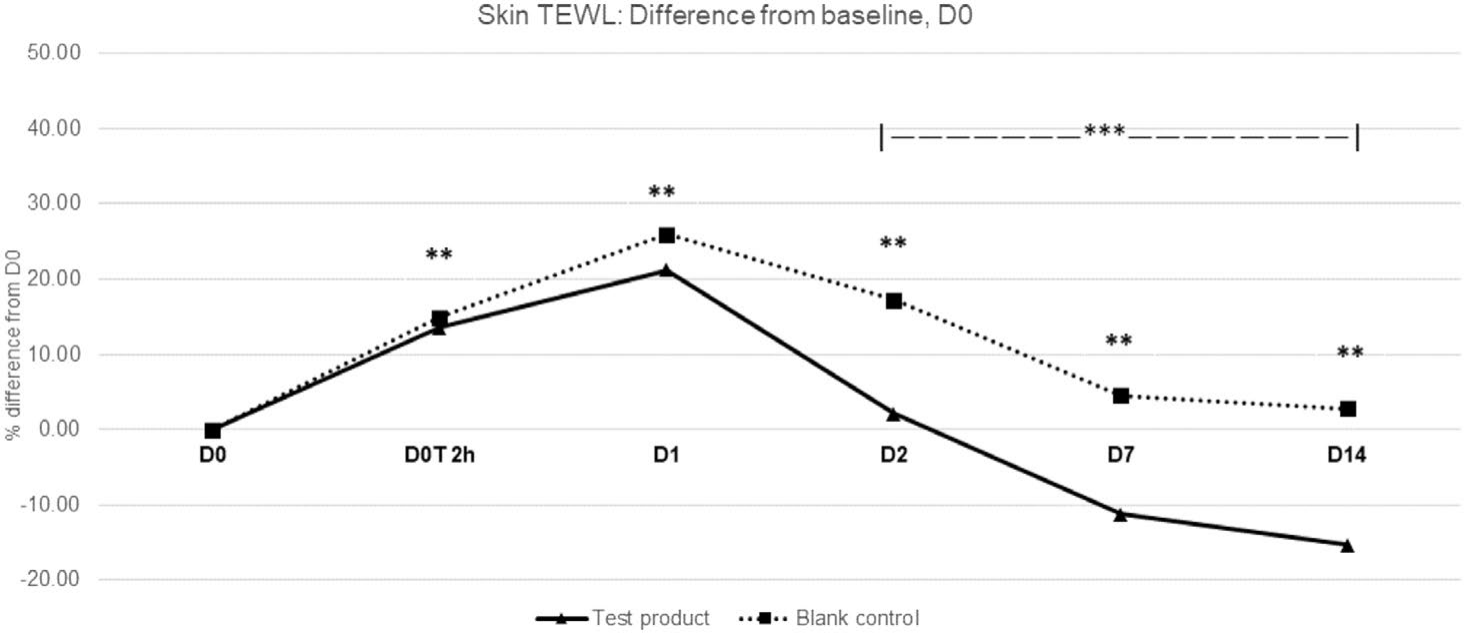
Intra‐group and inter‐group comparison of TEWL changes from baseline (D0) over a 14‐day period in a randomized clinical study (*n* = 35). TEWL (transepidermal water loss) was measured using a Tewameter. Skin TEWL percentage difference relative to baseline (D0) over 14 days was assessed using a Tewameter. Data represent the mean percentage change from baseline (D0T2h) for test product and blank control. **p < 0.01 indicates statistically significant differences versus baseline (D0T2h) in intra group comparisons; ***p < 0.001 indicates statistically significant differences between test product and blank control across the treatment period (D2–D14) in inter group comparisons.

**FIGURE 5:**
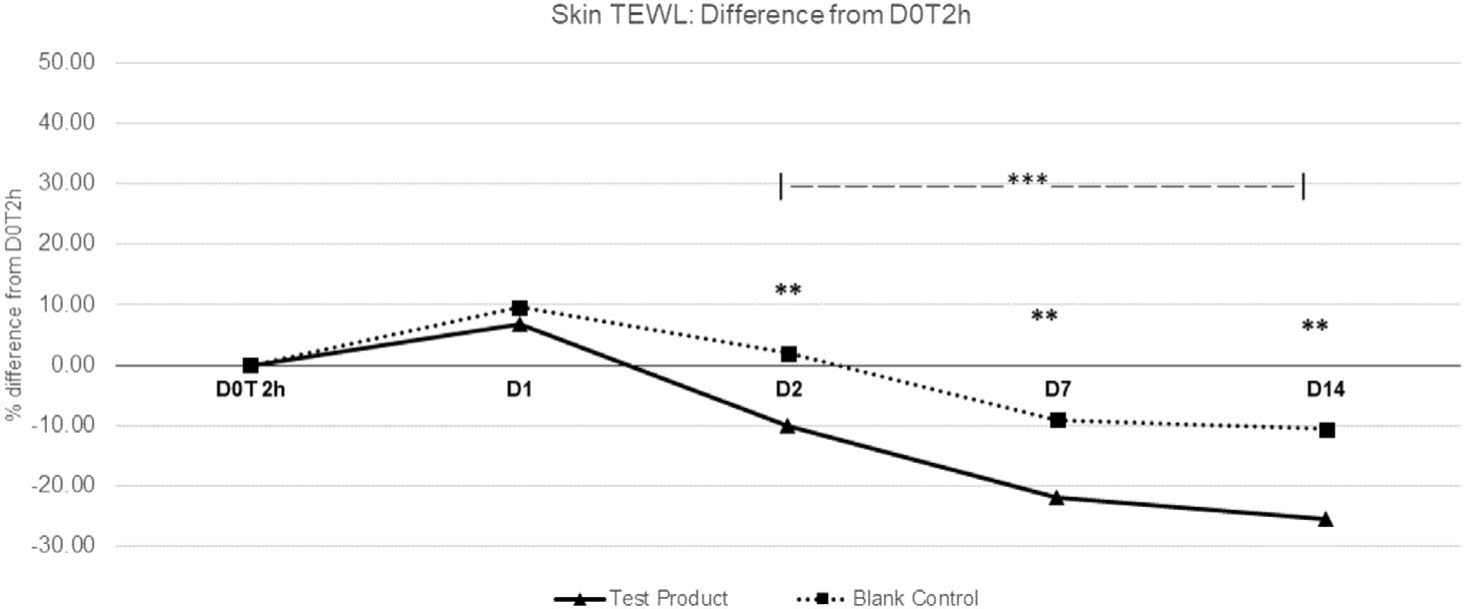
Intra‐group and inter‐group comparison TEWL changes from 2‐h post‐UV exposure (D0T2h) over a 14‐day period in a randomized clinical study (*n* = 35). TEWL (transepidermal water loss) was measured using a Tewameter. Skin TEWL percentage difference relative to baseline (to 2‐h post‐UV exposure (D0T2h)) over 14 days was assessed using a Tewameter. Data represent the mean percentage change from baseline (D0T2h) for test product and blank control. **p < 0.01 indicates statistically significant differences versus baseline (D0T2h) in intra group comparisons; ***p < 0.001 indicates statistically significant differences between test product and blank control across the treatment period (D2–D14) in inter group comparisons.

**TABLE 5:** Inter‐group comparison of transepidermal water loss (TEWL) changes from 2‐h post‐UV exposure (D0T2h) over a 14‐day period in a randomized clinical study (*n* = 35).

Skin TEWL (g/hm^2^)	D0T2h	D1	D2	D7	D14
*p*‐value (vs. D0) test product vs. blank control	0.156	0.145	< 0.001***	< 0.001***	< 0.001***
*p*‐value (vs. D0T2h) test product vs. blank control	N/A	0.294	< 0.001***	< 0.001***	< 0.001***

*Note:* TEWL was measured using a Tewameter. Values shown are *p*‐values comparing the test product with the blank control at each timepoint.

Abbreviations: D0, baseline; D0T2h, 2 h post‐UV exposure; D1, Day 1; D14, Day 14; D2, Day 2; D7, Day 7; TEWL, transepidermal water loss.

*** *p* < 0.001 indicates statistically significant inter‐group differences.

Inter‐group comparison showed significantly greater erythema reduction in the dual‐action sunscreen group versus blank control at D2, D7, and D14, both relative to baseline and D0T2h (*p* < 0.001) (Table 6). Dermatological clinical tolerance evaluation reported no edema, comedone, papules, or scaling in either group. No adverse events were reported.

**TABLE 6:** Inter‐group comparison of skin erythema from 2‐h post‐UV exposure (D0T2h) over a 14‐day period in a randomized clinical study (*n* = 35).

Skin TEWL (g/hm^2^)	D0T2h	D1	D2	D7	D14
*p*‐value (vs. D0) test product vs. blank control	0.925	0.346	< 0.001***	< 0.001***	< 0.001***
*p*‐value (vs. D0T2h) test product vs. blank control	N/A	0.730	< 0.001***	0.002	< 0.001***

*Note:* Skin erythema was measured using a Mexameter. Values shown are *p*‐values comparing the test product with the blank control at each timepoint.

Abbreviations: D0, baseline; D0T2h, 2 h post‐UV exposure; D1, Day 1; D14, Day 14; D2, Day 2; D7, Day 7.

*** *p* < 0.001 indicates statistically significant inter‐group differences.

#### 3.1.4 Barrier Repair: Study 4—Dual‐Action Sunscreen Restores Key Structural Proteins

In the UVB‐damaged reconstructed human epidermis model, compared with negative control (NC), the dual‐action sunscreen (test group) increased the expression of filaggrin, loricrin, and claudin1 levels by 273.68%, 139.39%, and 167.57%, respectively (Tables 7a, 7b, 7c). The barrier repair performance of the dual‐action sunscreen was similar to the positive control (PC), WY14643, an established barrier repair reference compound. Compared with NC, PC increased the expression of filaggrin, loricrin, and claudin1 levels by 263.16%, 124.24% and 164.86%, respectively. Antibody‐based immunofluorescence was observed under Olympus B× 43 fluorescence microscope. Representative fluorescence images are shown in Figure 6.

**TABLE 7a:** Filaggrin expression in UV‐irradiated (UVA 30 J/cm^2^ + UVB 50 mJ/cm^2^) 3D reconstructed human epidermis treated with dual‐action sunscreen for 4 days.

Group	Relative IOD	Standard deviation (SD)	*p*	Improvement vs. NC
BC	1.00	0.02	/	—
NC	0.38	0.03	0.000^##^	—
PC	1.38	0.08	0.000**	263.16%
Test group	1.42	0.04	0.000**	273.68%

*Note:* Protein expression was quantified by immunofluorescence and expressed as integrated optical density. Data are presented as mean ± SD. *p*‐values: *p* < 0.01^##^ vs. BC and *p* < 0.01** vs. NC.

Abbreviations: BC, blank control; FLG, filaggrin; IOD, integrated optical density; NC, negative control; PC, positive control; Test group, with dual‐action sunscreen.

**TABLE 7b:** Loricrin expression level in UV‐irradiated (UVA 30 J/cm^2^ + UVB 50 mJ/cm^2^) 3D reconstructed human epidermis treated with dual‐action sunscreen for 4 days.

Group	Relative IOD	Standard deviation (SD)	*p*	Improvement vs. NC
BC	1.00	0.062	/	—
NC	0.33	0.02	0.000^##^	—
PC	0.74	0.04	0.000**	124.24%
Test group	0.79	0.07	0.000**	139.39%

*Note:* LOR expression was quantified by immunofluorescence and expressed as integrated optical density. Data are presented as mean ± SD. *p*‐values: *p* < 0.01^##^ vs. BC and *p* < 0.01** vs. NC.

Abbreviations: BC, blank control; IOD, integrated optical density; LOR, loricrin; NC, negative control; PC, positive control; Test group, with dual‐action sunscreen.

**TABLE 7c:** Claudin‐1 expression level in UV‐irradiated (UVA 30 J/cm^2^ + UVB 50 mJ/cm^2^) 3D reconstructed human epidermis treated with dual‐action sunscreen for 4 days.

Group	Relative IOD	Standard deviation (SD)	*p*	Improvement vs. NC
BC	1.00	0.097	/	—
NC	0.37	0.03	0.00^##^	—
PC	0.98	0.01	0.00**	164.86%
Test group	0.99	0.03	0.00**	167.57%

*Note:* Protein expression was quantified by immunofluorescence and expressed as integrated optical density. Data are presented as mean ± SD. *p*‐values: *p* < 0.01^##^ vs. BC and *p* < 0.01** vs. NC.

Abbreviations: BC, blank control; CLDN1, claudin‐1; IOD, integrated optical density; NC, negative control; PC, positive control; Test group, with dual‐action sunscreen.

**FIGURE 6:**
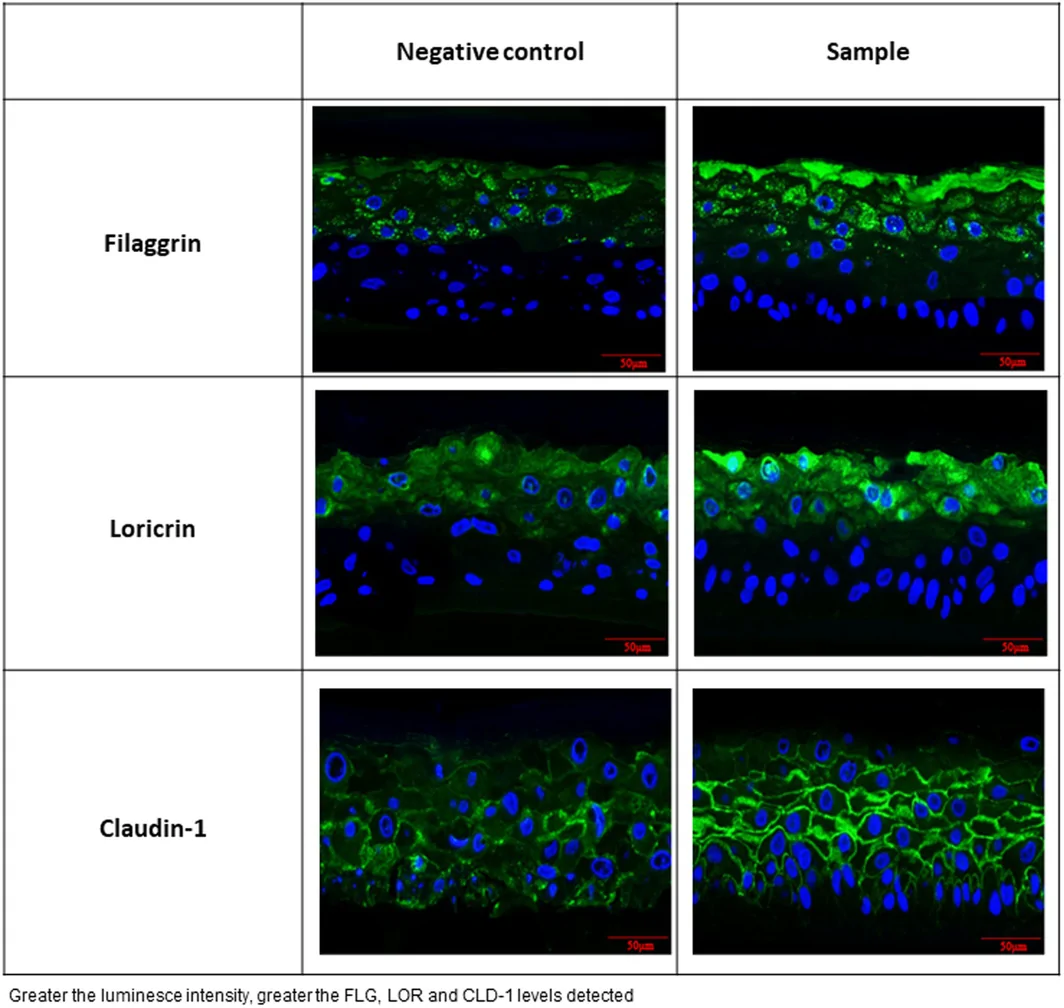
Immunofluorescence analysis of epidermal differentiation markers (FLG, LOR) and tight junction protein (claudin‐1) in UV‐irradiated 3D reconstructed human epidermis (UVA 30 J/cm^2^ + UVB 50 mJ/cm^2^) for 4 days. Images are representative of dual‐action sunscreen treated (sample) and negative control tissues. Fluorescence was measured using antibody‐based immunofluorescence, and the stained sections were observed under an Olympus BX43 fluorescence microscope. Green fluorescence intensity represents protein expression levels of filaggrin (FLG), loricrin (LOR), and claudin‐1 (CLDN1). Dual‐action sunscreen demonstrated significantly higher expression of these barrier‐related proteins compared to the control group.

### 3.2 Secondary Outcome

#### 3.2.1 Pigmentation and Skin Tone: Study 5—Dual‐Action Sunscreen Supports Pigment Recovery and Skin Tone Improvement

Compared with baseline, the dual‐action sunscreen group demonstrated statistically significant improvements in ITA° values at 1, 2, 3, and 4 weeks of use (all *p* < 0.001) (Figure 7). In contrast, the negative control group showed smaller but statistically significant increases over the same period. At all timepoints, improvements in ITA° were significantly greater in the dual‐action sunscreen group than in the negative control group (*p* < 0.001).

**FIGURE 7:**
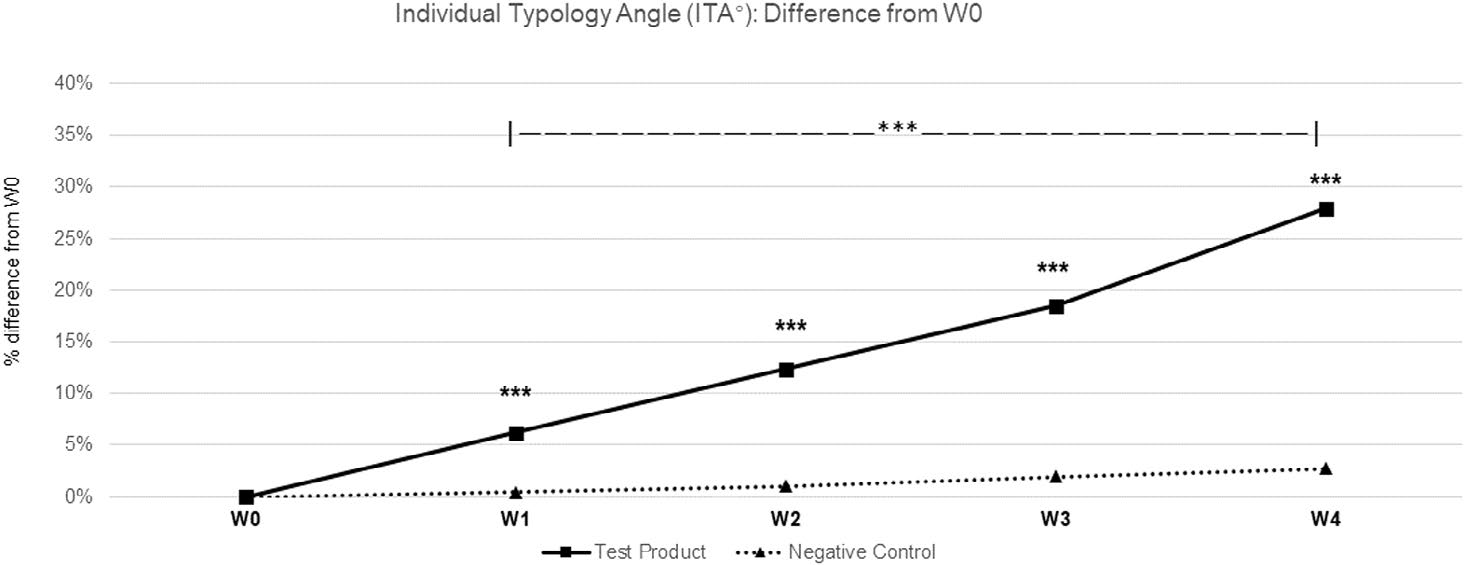
Comparative analysis of individual typography angle (ITA°) over 4 weeks in a human UV‐induced darkening model (*n* = 35). Data represent the percentage change in ITA° from Week 0 (W0) for test product and negative control at weekly interval W1–W4. ***p < 0.001 indicates statistically significant differences between the test product (dual‐action sunscreen) and negative control at the corresponding timepoints.

Skin melanin index (MI) values in the dual‐action sunscreen group decreased significantly from baseline at all evaluation time points (*p* < 0.001) (Figure 8). In contrast, the negative control group showed smaller reductions. The magnitude of MI reduction at all time points (*p* < 0.001) was significantly greater in the dual‐action sunscreen compared with the negative control.

**FIGURE 8:**
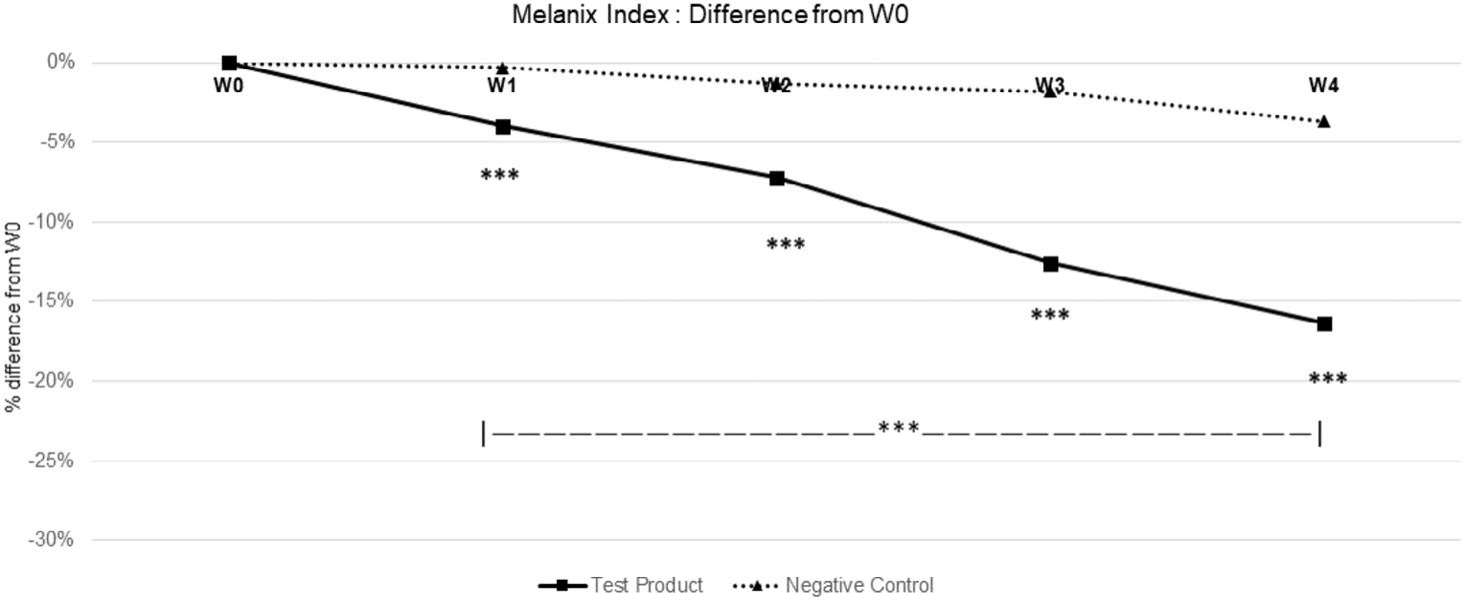
Comparative analysis of Melanin Index (MI) over 4 weeks in a human UV‐induced darkening model (*n* = 35). Data represent the mean percentage change Melanin Index from Week 0 (W0) for test product and negative control at weekly interval W1–W4. ***p < 0.001 indicates statistically significant differences between the test product (dual‐action sunscreen) and negative control at the corresponding timepoints.

Instrumental measurements of skin color parameters revealed significant improvement in skin brightness and visual skin color grading (*p* < 0.05) in the dual‐action sunscreen group compared with the negative control (Figures S1 and S2).

## 4 Discussion

Exposure to UVR is the primary contributor to photoaging, hyperpigmentation, and development of skin cancer. Regular sunscreen use is essential to mitigate both acute and chronic UV‐induced skin damage. While conventional sunscreens reduce the penetration of UVR into the skin, recent evidence shows subtle yet significant impairment in cellular repair and regeneration even with the use of SPF 30 formulation [8]. Consequently, cumulative photodamage, which starts accumulating as early as age 20, continuously undermines the strength and function of the skin barrier. Therefore, an optimal sunscreen should promote immediate photoprotection as well as barrier repair and recovery.

In this context, the present study demonstrates that this dual‐action sunscreen provides robust broad‐spectrum photoprotective performance. The dual‐action sunscreen achieved a mean SPF of 56.2, UVAPF of 18.9, and post‐immersion SPF of 36.0 corresponding to 63.9% water resistance, demonstrating its high photoprotective ability. From a clinical standpoint, maintaining an SPF of 36.0 after water exposure translates to blockade of over 97% of UVB radiation with effective photoprotection during 40 min of aquatic activities.

In addition, the formulation significantly reduced oxidative stress with a 57.74% reduction in ROS compared to negative control. This antioxidant action is especially relevant given the central role of oxidative stress in UV‐induced cellular damage and photoaging [21]. The highest ROS inhibition (77.38%) achieved by the positive control outperformed the dual‐action formulation. This was consistent with the expected benchmark ROS inhibition by the potent Vitamin C and Vitamin E combination [22]. However, the high antioxidant concentrations used in laboratory tests are unsuitable for cosmetic formulations due to chemical stability, rapid oxidation and skin irritation constraints. Consequently, the 57.74% ROS reduction successfully balances the antioxidant efficacy of the dual‐action sunscreen with chemical stability and dermatological safety. Furthermore, unlike the single‐target positive control, the dual‐action sunscreen provides a distinct clinical advantage through its multifunctional capabilities of photoprotection, antioxidant defense and barrier repair.

Beyond photoprotection, the dual‐action sunscreen demonstrated a marked ability to facilitate barrier recovery following UV exposure. Significant improvements in skin hydration and accelerated normalization of TEWL were observed on post‐UVR‐irritated skin, indicating barrier repair. Functional barrier recovery was observed through the upregulation of key epidermal barrier proteins, filaggrin, loricrin, and claudin‐1, which increased by 273.68%, 139.39%, and 167.57%, respectively, compared to negative control. Remarkably, the dual‐action sunscreen and WY14643, a potent PPARα agonist used as a positive control, resulted in statistically comparable upregulation of filaggrin, loricrin, and claudin‐1. This parity demonstrates the barrier restorative efficacy of the dual‐action sunscreen offering a significant clinical advantage combining robust photoprotection and potent skin barrier recovery. Restoration of these barrier proteins is a critical indicator of barrier repair capacity with previous literature establishing their role in barrier recovery [23, 24, 25].

Skin hydration and TEWL are well‐established non‐invasive indicators of barrier function, with increased TEWL and decreased hydration reflecting impaired barrier integrity [26]. In this study, skin hydration levels decreased immediately following UV exposure in both groups as expected; however, the dual‐action sunscreen group demonstrated a significant recovery and increase above baseline. In contrast, the blank control group showed decreased hydration at all post‐UV timepoints with no recovery to baseline. Similarly, following the acute post‐UV spike, TEWL in the blank control group remained elevated across all timepoints, whereas application of dual‐action sunscreen accelerated recovery of TEWL over time. These findings highlight that the dual‐action sunscreen strengthens barrier function and enhances barrier recovery following UV‐irritation.

The improvement in epidermal barrier function can be attributed to the inclusion of hydrolysed hyaluronic acid, panthenol, and vitamin C within the dual‐action sunscreen. A recent international expert consensus on integrated skincare active ingredients highly ranked hyaluronic acid, panthenol, and vitamin C for their barrier repair, healing, and anti‐inflammatory properties [27]. Each of these ingredients has independently demonstrated their ability to strengthen and support barrier function, thereby justifying the rationale for their inclusion in the dual‐action sunscreen. Hyaluronic acid (HA) is well recognized for improving skin hydration, with hydrolysed forms allowing enhanced penetration to mitigate photoaging [28]. Panthenol, a precursor of vitamin B5, is a key contributor to barrier repair and recovery through its ability to reduce TEWL, improve hydration, and alleviate inflammation, particularly in sensitive skin [29]. Vitamin C further improves barrier function by promoting keratinocyte differentiation, increasing ceramide and barrier lipid production, protecting against oxidative damage, and reducing inflammatory response [30]. The combined actions of these ingredients are consistent with the structural and functional barrier improvements reported in this study.

The dual‐action sunscreen formulated using the Smart Layers technology adopts a multilayered approach by integrating broad spectrum UV filtration with skin supportive active ingredients. Smart Layers Technology consists of an outer layer and an inner layer, where the outer layer with UV filters remains on the skin surface preventing UV penetration, while the inner layer delivers active ingredients to the epidermis enabling active recovery. The outer layer of Smart Layers technology is designed to deliver stable, broad‐spectrum protection against both UVA and UVB radiation. The combination of selective organic and inorganic UV filters provides coverage across the 290–400 nm range using photostable combinations of organic and inorganic filters. The inner layer incorporates barrier supportive, antioxidative, anti‐inflammatory, and pigment‐modulating components that mitigate photodamage. Sclareolide has been shown to inhibit inflammatory signaling and melanocyte activation triggered by environmental stressors, while phenylethyl resorcinol reduces melanin synthesis through tyrosinase inhibition [31, 32, 33]. This layered design enables simultaneous photoprotection and skin recovery.

Traditional sunscreens mainly focus on preventing UV penetration, often overlooking the importance of barrier repair. The present study addresses this gap, particularly in Asian populations with distinct phototypes and increased susceptibility to photopigmentary disorders and late‐stage melanoma. Despite these risks, effective and consistent adoption of photoprotective behaviors remains limited [17]. A dual‐action sunscreen that combines immediate photoprotection and long‐term skin barrier repair represents a clinically relevant intervention to improve user compliance and photoprotective outcomes. Future observational studies to assess whether use of dual‐action sunscreen leads to long‐term improvements in photoprotection habits among individuals with lower compliance will be insightful.

## 5 Strengths and Limitations

This study has several strengths, including the use of complementary methodologies to establish the photoprotective efficacy, structural and functional barrier recovery, antioxidant and pigment modulating efficacy of the dual‐action sunscreen. However, certain limitations should be acknowledged. The longest study duration was 4 weeks, and studies with longer follow‐ups (at least 6 months) are required to establish sustained benefits. We acknowledge that a vehicle‐only sunscreen control was not included in this study because the primary objective was to evaluate the efficacy of the finished formulation as a whole rather than isolate the contribution of individual active ingredients. This study did not include a long‐term safety or comedogenicity assessment. The safety evaluation was based on a combination of a 14‐day usage, a 4‐week consumer use study, and Human Repeat Insult Patch Testing (HRIPT), all of which demonstrated favorable tolerability with no significant safety concerns observed during the respective study periods. In addition, a 4‐week human UV‐induced pigmentation recovery study was conducted, which demonstrated continued product tolerability throughout the study duration. Although this does not constitute a long‐term safety assessment, it provides additional evidence supporting short to medium‐term use. Longer‐duration studies would be valuable to further confirm long‐term safety and real‐world use over extended periods.

This study did not directly measure serum vitamin D levels, which represents a limitation. However, concerns that high SPF sunscreens may reduce vitamin D synthesis should be weighed against the established benefits of photoprotection. A recent meta‐analysis of randomized controlled trials found that sunscreen use was associated with a modest reduction in serum 25‐hydroxyvitamin D concentrations (approximately 2 ng/mL), although the clinical significance of this effect remains uncertain and requires further research [34]. Moreover, evidence suggests that other photoprotective practices, particularly protective clothing and shade‐seeking behaviors, have a greater impact on vitamin D status than sunscreen use alone [35]. Moreover, under real‐world conditions, sunscreen is rarely applied at the standardized thickness used for SPF testing (2 mg/cm^2^), which may further limit its effect on cutaneous vitamin D synthesis [36]. Thus, while vitamin D status was not assessed, the available evidence suggests that the photoprotective benefits of regular sunscreen use substantially outweigh any potential impact on vitamin D synthesis in real world settings.

## 6 Conclusion

Cumulative photodamage and UV‐induced molecular perturbations in cellular repair mechanisms continuously weaken the skin barrier. While conventional sunscreens provide photoprotection, they do not address skin barrier compromise. The dual‐action sunscreen (SPF 50+/PA++++) bridges this therapeutic gap by combining photoprotection and skin barrier recovery, shifting the paradigm from passive protection to active defense against photoaging.

## Funding

This study was funded by Kenvue APAC, JNTL Consumer Health K.K. (Japan).

## Ethics Statement

The study was approved by the Guangdong Daily Chemical Institutional Review Board (IRB) Ethics Committee.

## Consent

Informed consent was obtained from all participants.

## Conflicts of Interest

Authors Jiamin Gu, Yue Ying Fu, Vanessa Koh, Hiroki Fujita, Jessica Wang, Sheila Ching Chua, Yuan Yuan Duan, Daiki Arai, Junchao Zhang and Mao Kasahara are employees of Kenvue at the time of manuscript writing. Wei Lai is an advisor to Kenvue APAC, and the principal consultant of this study. The authors report no other conflicts of interest in this work.

## Supporting information


**Table S1:** Study 1—In vivo photoprotection study design.
**Table S2:** Study 2—Ex vivo antioxidant (ROS) study design.
**Table S3:** Study 3—Barrier repair randomized clinical trial.
**Table S4:** Study 4—In vitro epidermal barrier protein expression study design.
**Table S5:** Study 5—In vivo UV‐induced pigmentation study design.
**Figure S1:** Comparative analysis of L* values (brightness) over 4 weeks in a human UV‐induced darkening model (*n* = 35). Data represent the mean percentage change in L* values (brightness) from Week 0 (W0) for test product and negative control at weekly interval W1–W4. ***p < 0.001 indicates statistically significant differences between the test product (dual‐action sunscreen) and negative control at the corresponding timepoints.
**Figure S2:** Comparative analysis of a* values (redness) over 4 weeks in a human UV‐induced darkening model (*n* = 35). Data represent the mean percentage change in a* values (redness) from Week 0 (W0) for test product and negative control at weekly interval W1–W4. **p < 0.01 indicates statistically significant differences from baseline and between the test product (dual‐action sunscreen) and negative control at the corresponding timepoints.

## Data Availability

The data that support the findings of this study are available from the corresponding author upon reasonable request.

## Endnotes

^1^
WR_i_: individual percentage of water resistance; s: standard deviation; n: total number of subjects in test; t_u_: *t* value from the one‐sided student‐*t* distribution at a probability level *p* = 0.10 with *n*‐1 degrees of freedom.
