# Supplementary material for: Efficacy of a Dual‐Action SPF 50+/PA++++ Sunscreen: A Multi‐Targeted Approach to Photoprotection and Skin Barrier Recovery
Source: J Cosmet Dermatol. 2026 Oct 22;25(10):e71253. doi: 10.1111/jocd.71253 (PMC13691168; doi:10.1111/jocd.71253)
Supplement: Supplementary file 1 [file JOCD-25-e71253-s001.docx]

# **[Supplementary Material]**

**Table S1. Study 1 – *In vivo* photoprotection study design**

| **Element** | **Description** |
| --- | --- |
| Population | Healthy adults (18–60 years) |
| Design | Single‑center, evaluator‑blinded, self‑controlled |
| UV Source | Solar simulator (UVA + UVB) |
| Test Area | Infrascapular back |
| Intervention | Dual‑action sunscreen (2 mg/cm²) |
| Comparator | Unprotected skin; No separate positive or negative product controls |
| Endpoints | SPF, UVAPF, water resistance |

*Design and methodology of the in vivo photoprotection study evaluating the sunscreen’s ultraviolet B (UVB) and ultraviolet A (UVA) protective efficacy.*
This single‑center, evaluator‑blinded, self‑controlled clinical study assessed sun protection factor (SPF), UVA protection factor (UVAPF), and water resistance following controlled UVA and UVB exposure in healthy adult volunteers. Outcomes were determined by comparing sunscreen‑protected skin with unprotected control sites.

**Table S2. Study 2 – *Ex* vivo antioxidant (ROS) study design**

| **Element** | **Description** |
| --- | --- |
| Model | *Ex* vivo human skin explants |
| UV Exposure | UVA (30 J/cm²) + UVB (50 mJ/cm²) |
| Comparators | 1. Blank control (BC; no UV exposure or treatment), 2. Negative control (NC; UV exposure without treatment) 3. Positive control (PC; UV exposure with antioxidant treatment) 4. Test group (UV exposure with dual‑action sunscreen application at 2 mg/cm²). |
| Intervention | Dual‑action sunscreen (2 mg/cm²) |
| Endpoint | ROS levels (DCFH‑DA fluorescence, IOD)  Fluorescence intensity visualized with Olympus BX43 |

*Design of the ex vivo human skin explant model used to evaluate antioxidant efficacy following ultraviolet radiation exposure.*
Human skin explants were exposed to combined UVA and UVB irradiation and assigned to blank control, negative control, positive control, or test product groups. Antioxidant efficacy was assessed by quantifying reactive oxygen species (ROS) levels using fluorescence‑based integrated optical density measurements.

**Table S3. Study 3 – barrier repair randomized clinical trial**

| **Element** | **Description** |
| --- | --- |
| Design | Randomized, evaluator‑blinded controlled trial |
| Participants | 35 eligible participants |
| Comparators | 1. Test group with dual‑action sunscreen (intervention) 2. Blank control (untreated or vehicle‑treated site) |
| UV Exposure | Controlled UV irritation |
| Test area | Skin on the back |
| Intervention | Daily application for 14 days post‑UV |
| Endpoints | Skin hydration, TEWL, clinical tolerance |

*Design of the randomized, evaluator‑blinded clinical study assessing post‑UV skin barrier repair.*
Participants underwent controlled UV‑induced skin irritation, followed by daily application of the dual‑action sunscreen or blank control. Skin hydration, transepidermal water loss (TEWL), and clinical tolerance were evaluated to assess functional skin barrier recovery.

**Table S4. Study 4 – In vitro epidermal barrier protein expression study design**

| Element | Description |
| --- | --- |
| Model | UVB‑damaged 3D reconstructed human epidermis |
| UV Dose | UVB 600 mJ/cm² |
| Comparators | 1. Blank control (BC; no UV exposure) 2. Negative control (NC; UV exposure without treatment) 3. Positive control (PC; UV exposure with reference barrier‑repair compound) 4. Test group (UV exposure with topical application of dual‑action sunscreen) |
| Intervention | Dual‑action sunscreen (2 mg/cm²) |
| Endpoints | FLG, LOR, CLDN1 expression (IOD)  Fluorescence intensity visualized with Olympus BX43 |

*Design of the in vitro UVB‑damaged reconstructed human epidermis model evaluating epidermal barrier protein expression.*
A three‑dimensional human epidermal model subjected to UVB irradiation was used to quantify changes in filaggrin (FLG), loricrin (LOR), and claudin‑1 (CLDN1) expression following treatment with the dual-action sunscreen, relative to negative and positive controls.

**Table S5. Study 5 – In vivo UV‑induced pigmentation study design**

| Element | Description |
| --- | --- |
| Population | Healthy adults (21–60 years) |
| UV Model | Repeated UV‑induced skin darkening |
| Comparators | 1. Test group (with dual‑action sunscreen) 2. Blank control (untreated site), 3. Positive control (7% ascorbic acid) |
| Endpoints | Skin brightness (L*), redness (a*), ITA°, melanin index |

*Design of the in vivo ultraviolet radiation‑induced pigmentation model assessing skin tone and pigmentation recovery.*
Healthy adult subjects underwent repeated UV exposure to induce skin darkening, followed by four weeks of daily product application. Skin brightness, redness, and overall skin tone were evaluated using colorimetric parameters (L*, a*) and Individual Typology Angle (ITA°) to assess pigmentation improvement.

**SUPPLEMENTARY FIGURES**

**Figure S1: Comparative analysis of L* values (brightness) over 4 weeks in a human UV‑induced darkening model (n = 35)**

Data represent the mean percentage change in L* values (brightness) from week 0 (W0) for Test Product and Negative Control at weekly interval W1-W4. ***p < 0.001 indicates statistically significant differences between the test product (dual-action sunscreen) and negative control at the corresponding timepoints.

**Figure S2: Comparative analysis of a* values (redness) over 4 weeks in a human UV‑induced darkening model (n = 35)**

Data represent the mean percentage change in a* values (redness) from week 0 (W0) for Test Product and Negative Control at weekly interval W1-W4.

**p < 0.01 indicates statistically significant differences from baseline and between the test product (dual-action sunscreen) and negative control at the corresponding timepoints.
